# Linked Lives: How Homebound Status Shapes Physical Health Interdependence in Care Dyads

**DOI:** 10.1093/geroni/igaf122.1254

**Published:** 2025-12-31

**Authors:** Hyojin Choi, Kate Perepezko

**Affiliations:** University of Vermont, Burlington, Vermont, United States; Miami University, Oxford, Ohio, United States

## Abstract

Homebound older adults - those who cannot leave home without someone’s help – tend to have multiple health conditions, substantial functional limitations, and complex care needs. Family caregivers play an instrumental role in supporting these adults and may experience greater physical strain and an increased risk of frailty. Given the strong connection between caregiver and older adult health outcomes, their frailty may also be interdependent. The current study examined this interdependence and if it varied by older adult homebound status. Using dyadic data from the National Health and Aging Trends Study and the National Survey of Caregiving (2015 and 2017), the sample was divided into three groups: becoming homebound (n = 108; not homebound in 2015, homebound in 2017), long-term homebound (n = 235; homebound in 2015 and 2017) and never homebound (n = 166; not homebound in 2015 or 2017). Actor-Partner Interdependence models (APIM) were employed to examine the interdependence of frailty in caregiving dyads. In long-term homebound and never homebound dyads, APIM models revealed partner effects from caregiver to care recipient, indicating when caregivers were frail at baseline, frailty of care recipients declined in the following wave. Interestingly, in becoming homebound dyads, a care recipient to caregiver partner effect was observed, with baseline care recipients’ frailty associated with subsequent caregiver frailty. These preliminary findings demonstrate how frailty interdependence can vary by homebound status. These findings have implications for creating interventions aiming to prevent and reduce frailty for older adults and their caregivers, suggesting dyadic interventions may have greater success.

